# Somatostatin Derivate (smsDX) Attenuates the TAM-Stimulated Proliferation, Migration and Invasion of Prostate Cancer via NF-κB Regulation

**DOI:** 10.1371/journal.pone.0124292

**Published:** 2015-05-26

**Authors:** Zhaoxin Guo, Zhaoquan Xing, Xiangyu Cheng, Zhiqing Fang, Chao Jiang, Jing Su, Zunlin Zhou, Zhonghua Xu, Anders Holmberg, Sten Nilsson, Zhaoxu Liu

**Affiliations:** 1 Department of Urology, Qilu Hospital of Shandong University, Jinan, Shandong, China; 2 The Key Laboratory of Cardiovascular Remodeling and Function Research, Chinese Ministry of Education and Chinese Ministry of Public Health, Department of Cardiology, Qilu Hospital of Shandong University, Ji’nan, Shandong, China; 3 School of Nursing, Shandong University, Jinan, Shandong, China; 4 Department of Oncology and Pathology, Karolinska Institutet, Stockholm, Sweden; Istituto Superiore di Sanità, ITALY

## Abstract

Tumor development and progression are influenced by macrophages of the surrounding microenvironment. To investigate the influences of an inflammatory tumor microenvironment on the growth and metastasis of prostate cancer, the present study used a co-culture model of prostate cancer (PCa) cells with tumor-associated macrophage (TAM)-conditioned medium (MCM). MCM promoted PCa cell (LNCaP, DU145 and PC-3) growth, and a xenograft model in nude mice consistently demonstrated that MCM could promote tumor growth. MCM also stimulated migration and invasion *in vitro*. Somatostatin derivate (smsDX) significantly attenuated the TAM-stimulated proliferation, migration and invasion of prostate cancer. Immunohistochemistry revealed that NF-κB was over-expressed in PCa and BPH with chronic inflammatory tissue specimens and was positively correlated with macrophage infiltration. Further investigation into the underlying mechanism revealed that NF-κB played an important role in macrophage infiltration. SmsDX inhibited the paracrine loop between TAM and PCa cells and may represent a potential therapeutic agent for PCa.

## Introduction

Prostate cancer (PCa) is the most common cancer affecting men and represents the second leading cause of cancer related mortality in the western world[1]. The majority of prostate cancer progresses from prostatic intraepithelial neoplasia through locally invasive adenocarcinoma to castration resistant prostate cancer (CRPC)[2], which leads to the high mortality and little treatment is effective. Despite a lot of research, the intrinsic mechanism about CRPC is still unclear. Recently, zhu et al found that cytokines including IL-1β produced by macrophages contributed to the progression of CRPC[3].

Macrophages are usually the most abundant immune population present in the tumor micro-environment[4–6]. Recently, two distinct states of polarized macrophages have been identified: the ‘classically’ activated (M1) and the ‘alternatively’ activated (M2) macrophages. And it is now generally accepted that TAM have an M2 phenotype and could produce a series of cytokines/chemokines in the local tumor microenvironment that might be associated with promotion of tumor growth, angiogenesis, and metastasis in prostate cancer[7–10]. However, the detailed mechanism remains unknown.

Nuclear factor-κB (NF-κB) is a member of a family of ubiquitously expressed transcription factors that participates in inflammation, immunity and oncogenesis[11,12]. It is widely present in most (if not all) cells, including macrophages. And its aberrant activation has also been observed in most tumors. Recently several studies have shown that cytokines (e.g., IL-1 and TNF) produced by TAM could promote the activation of NF-κB[13,14], then promote tumor cell proliferation, tumorigenesis, migration and invasion[15,16]. In addition, activation of NF-κB in macrophages could exacerbate insulin insensitivity and affect glucose metabolism, and involves in metabolic disorders including obesity, insulin resistance, type 2 diabetes, and atherosclerosis[17–19]. It is believed that NF-kB promotes cell growth and oncogenesis, at least in part, by reorganizing metabolic circuitries.

Somatostatin (sms) is an acidic polypeptide, which is widely distributed throughout the central nervous system, different peripheral tissues and organs. It has a wide range of biological activities in endocrine, inflammation and tumor. Sms derivative (smsDX) is based on natural sms14, and could bind to all five somatostatin receptor subtypes (SSTR)[20]. Our previous research has found smsDX could promote apoptosis, suppress the proliferation, migration and invasion of prostate cancer cells[21]. And several studies showed somatostatin receptor was present in macrophages, sms and its analogue could modulate the function of macrophages[22–24]. However, the mechanism remains unclear. Somatostatin and its analogue have been reported in several studies to suppress the activity of NF-κB[25,26]. However, whether smsDX can regulate NF-κB in prostate cancer remains unknown.

The present study assessed the correlation between NF-κB expression and TAM infiltration in prostate cancer (PCa), benign prostatic hyperplasia (BPH) and BPH with chronic inflammation specimens via immunohistochemistry. To simulate the tumor microenvironment, a co-culture model of PCa cells (LNCaP, DU145 and PC-3) with TAM-conditioned medium (MCM) were established *in vitro*. MCM promoted the proliferation, migration and invasion of PCa cells, and this simulation could be inhibited by smsDX. Further investigation indicated that NF-κB played a vital role in this process, and may be involved in an autocrine/paracrine loop between TAMs and PCa cells. Our findings suggest that an inflammatory microenvironment promotes PCa progression, and that smsDX may represent an auxiliary drug for prostate cancer due to its potential anti-inflammatory role.

## Results

### P65 expression is positively correlated with macrophage recruitment in human prostate tissues

Immunoreactive staining for the P65 subunit of NF-κB was assessed in the specimen of 24 PCa, 12 BPH with chronic inflammation and 33 BPH specimens. When the extent of staining in all of the tissue specimens was quantified on the 0–3+ scale, we observed a graded increase among the BPH tissues, BPH with chronic inflammation specimens, and PCa. None of the BPH tissues stained as 3+, and 87.8% exhibited 0+ or 1+ staining. The amount of staining in the BPH with chronic inflammation specimens was intermediate, with 75% of specimens exhibiting 1+ or 2+ staining and 25% exhibiting 3+. In contrast, all of the cancer specimens were 2+ to 3+ (37.5 and 62.5%, respectively), as shown in Table 1. Additionally, only weak P65 staining was observed in the cytoplasm in BPH tissues, P65 staining was observed in both the cytoplasm and nucleus of tumor and inflammatory cells.

**Table 1:** Characteristics of patients with PCa and the association between NF-κB expression and clinicopathologic variables.

Variable		NF-KB staining	
	Total	2+	3+
Age, years (median 71)			
<71	11	5	6
≥71	13	4	9
Gleason score			
<7	7	3	4
= 7	7	4	3
>7	10	2	8
T stage			
T_1,2_	21	9	12
T_3,4_	3	0	3
N stage			
N_0_	21	9	12
N_1_	3	0	3
M stage			
M_0_	16	8	8
M_1_	8	1	7
TNM stage			
Ⅰ-Ⅱ	15	8	7
Ⅲ-Ⅳ	9	1	8

Abbreviation: NF-κB nuclear factor kappa-B; PCa Prostate cancer; 2, moderate staining; 3, strong staining.

**Fig 1:**
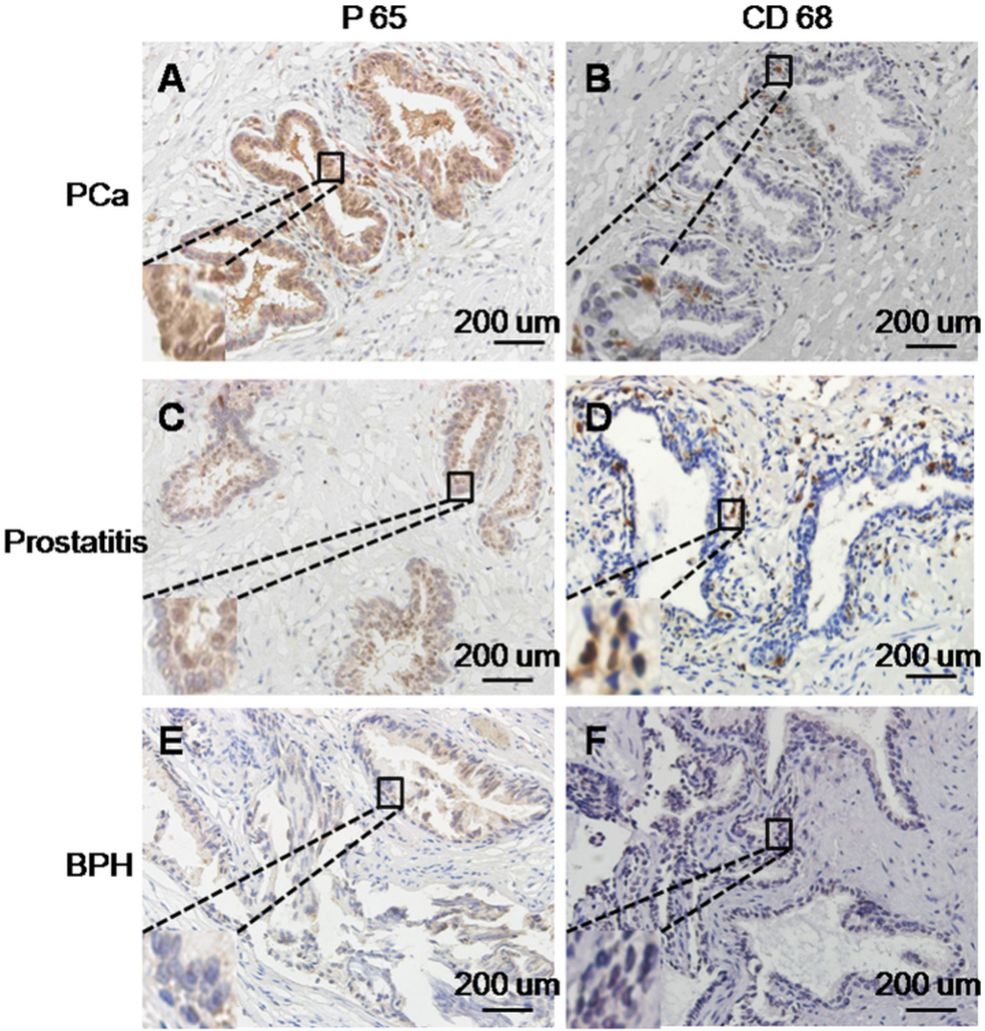
Immunohistochemical staining of NF-κB and CD68 in PCa, prostatitis and BPH specimens. (A) Strong staining of NF-κB in the cytoplasm and nucleus of PCa. (B) Moderate infiltration of macrophages in PCa. (C) Moderate staining of NF-κB in the cytoplasm and nucleus of prostatitis. (D) High infiltration of macrophages in chronic prostatitis. (E) Weak staining of NF-κB in the cytoplasm of BPH. (F) Mild infiltration of macrophages in BPH. All images are representative images.

To further assess the relationship between P65 and macrophage infiltration in human prostate tissues, unstained sections were immunolabeled with CD68. As shown in Fig 1, CD68-positive cells and P65 were coordinately expressed at moderate to high levels in BPH with chronic inflammation and prostate cancer lesions. In contrast, minimal CD68 staining was observed for BPH. Moreover, the majority of the CD68-positive cells exhibited morphological features of macrophages. Therefore, P65 expression was positively correlated with macrophage density.

### SmsDX inhibited the MCM-promoted proliferation and colony formation of prostate cancer cells

**Fig 2:**
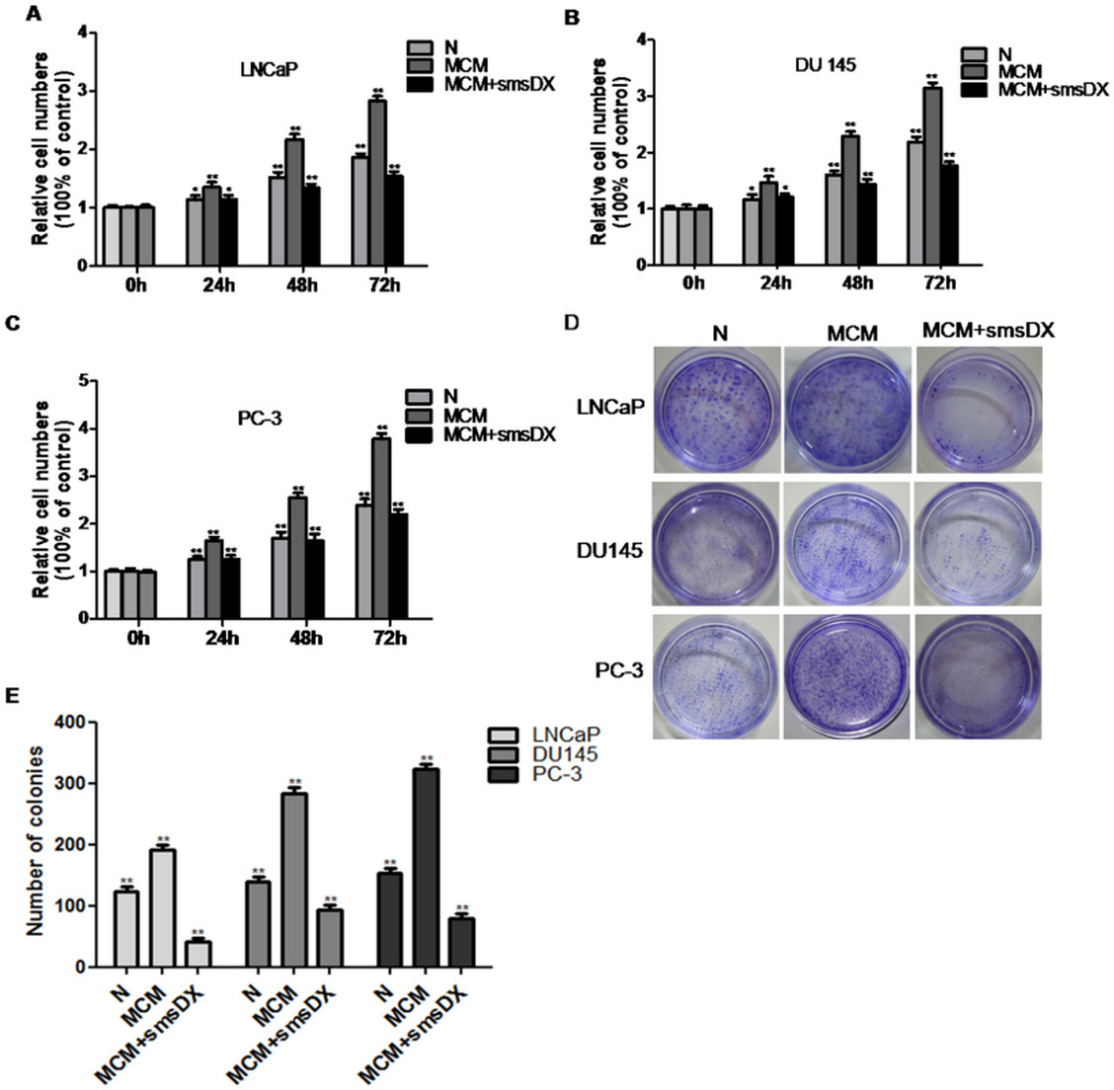
SmsDX suppresses the stimulative effect of MCM on the cell viability of LNCaP, DU145 and PC-3 cells. The effect of MCM and/or smsDX on cell viability was measured via CCK-8 assay. (A) LNCaP cells, (B) DU145 cells and (C) PC-3 cells were co-cultured with MCM and/or treated with smsDX for 24, 48 and 96 h. Co-culture with MCM significantly promoted the proliferation of PCa cells in a time-dependent manner, wheras smsdx could substantially attenuate this influence. (D) The effect of MCM and smsDX on colony formation. (E) The number of colonies, defined asclusters of at least 50 cells, was counted under microscope. The results represent means ± SD of three independent experiments. Data are presented as the means ± SD, *p<0.05, **p<0.01.

To study the effects of MCM and smsDX on the proliferation of PCa cells, LNCaP, DU145 and PC-3 cells were separately exposed to MCM and smsDX for 24, 48 and 72 h in a CCK-8 assay. Consequently, MCM significantly promoted the proliferation of LNCaP, DU145 and PC-3 cells in a time-dependent manner, whereas smsDX could weaken the impact of MCM on cell proliferation (Fig 2). The viability of LNCaP, DU145 and PC-3 cells increased to 51.7%, 44.3% and 57.6%, respectively, after exposure to MCM for 72 h, however, viability decreased to 44.8%, 44.1% and 41.6%, respectively, upon smsDX treatment.

We further examined the effects of MCM and smsDX on the colony formation of PCa cells. MCM dramatically facilitated the colony formation of LNCaP, DU145 and PC-3 cells. However, the colony number was significantly decreased after treatment with smsDX as shown in Fig 2 (*P<0.05, **P<0.01).

### Effects of MCM and smsDX on cell migration and invasion *in vitro*

**Fig 3:**
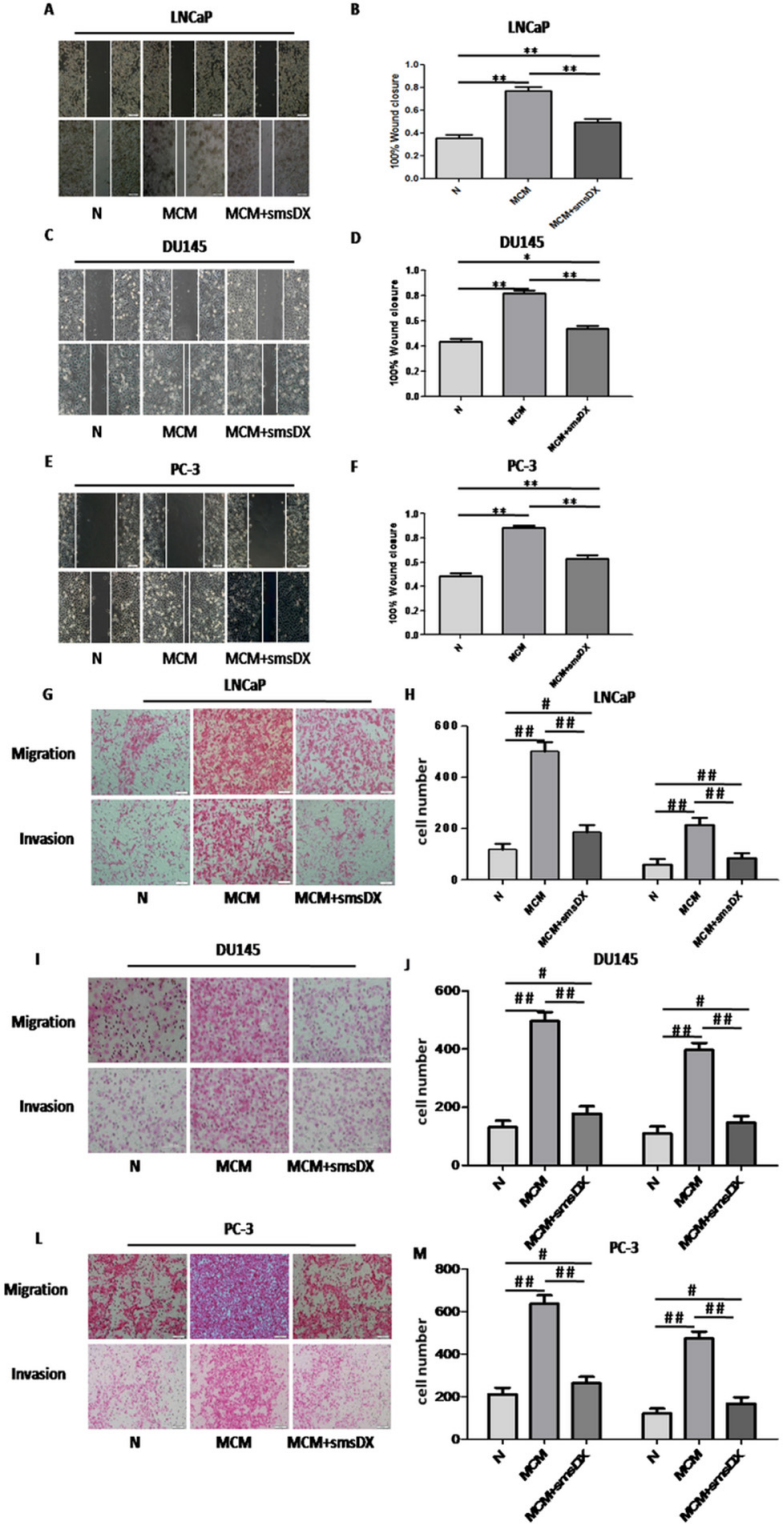
The enhanced cell migration and invasion induced by MCM were inhibited by smsDX. (A, C, E) Co-culture with MCM enhanced the cell migration of LNCaP, DU145 and PC-3 cells. SmsDX suppressed the effect of MCM. (B, D, F) The migration change is presented as the percentage of the initial distance between the two edges. (G, I, L) Transwell migration and invasion assay of LNCaP, DU145 and PC-3 cells treated with MCM and/or smsDX. (H, J, M) The LNCaP, DU145 and PC-3 cells that successfully migrated and invaded were counted. Data are presented as the means ± SD of three independent experiments. Data are presented as mean ± SD, *p<0.05, **p<0.01,#p<0.05,## p<0.01.

Scratch assays were performed to evaluate the effects of MCM and smsDX on the migration of PCa cells. The migration capabilities of LNCaP, DU145 and PC-3 were markedly increased after co-culture with MCM for 24 h (Fig 3). To further test the influence of MCM and smsDX on cell migration and invasion, transwell assay was performed. Our results demonstrated that the number of migratory and invasive cells passing through the filter increased significantly when macrophages were cultured in the lower chamber (Fig 3) (#P<0.05, ##P<0.01). However, smsDX (10 nM) could dramatically inhibit the migration and invasion of PCa cells.

### SmsDX inhibits MCM-dependent tumor growth in a xenograft model

**Fig 4:**
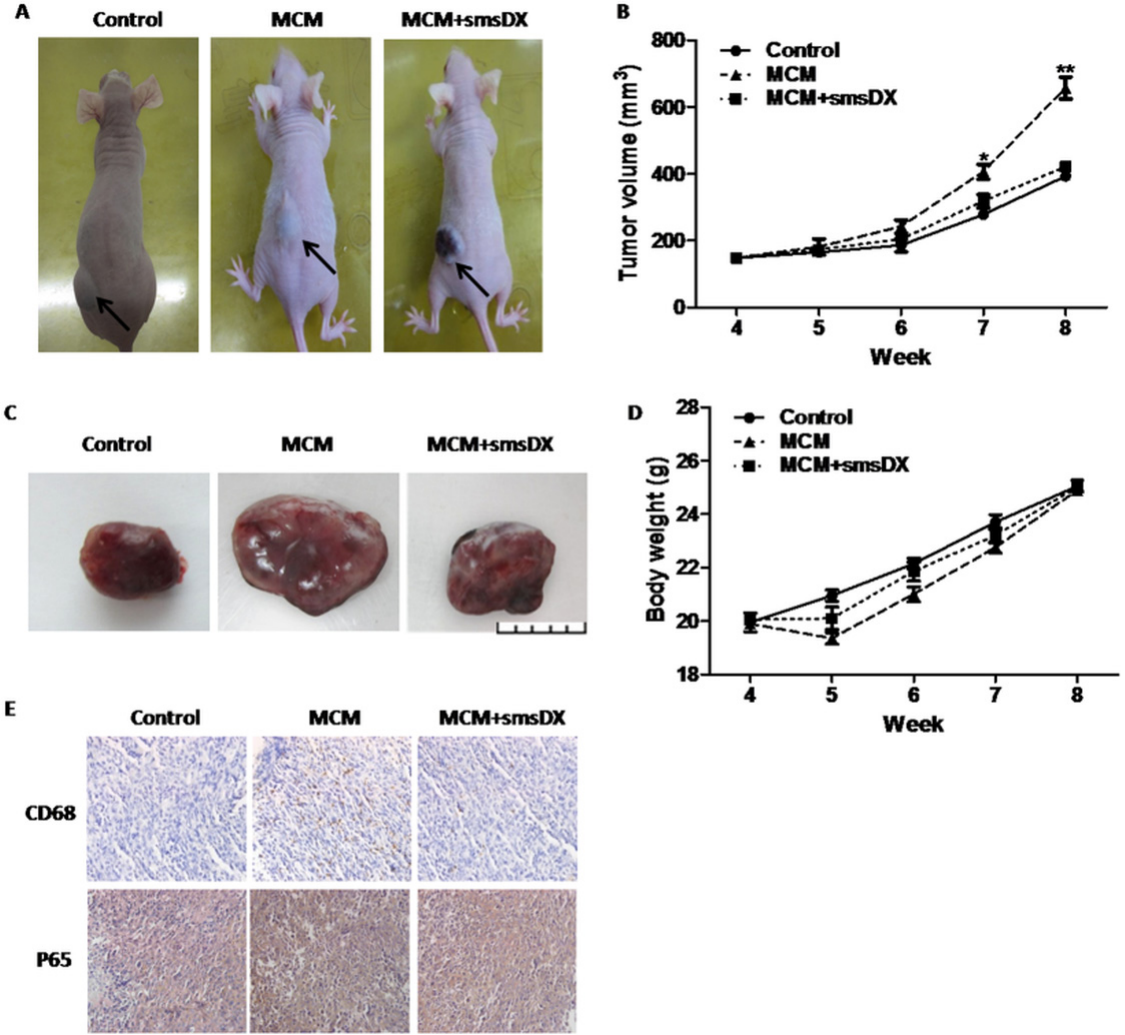
Co-culture with MCM promoted growth by activating NF-κB and smsDX attenuated this effect in PCa tumor xenografts. Images of the nude mice (A) and the excised tumors (C) were taken from different groups. (B) Body weight curve of nude mice bearing PC-3 tumors in different groups. (D) Graphs representing the average tumor volumes of PC-3 xenografts treated with MCM and/or smsDX. (E) The correlation between CD68 and NF-κB in PCa tumor xenografts. Data are presented as the means ± SD, *p<0.05, **p<0.01.

After demonstrating the efficacy of smsDX in inhibiting PCa cell viability and invasion *in vitro*, we further investigated its potential antitumor effect in mice. PC-3 cells (5×10^6^) were injected subcutaneously into each flank of nude mice. Treatment with MCM significantly increased the average tumor volume. However, tumor growth was inhibited after treatment with smsDX at 1 mg/kg/d (Fig 4) (*P<0.05, **P<0.01). Furthermore, no significant toxicity was observed in mice treated with MCM and smsDX (1 mg/kg/d) as evaluated using the weight of each animal in the 3 groups (Fig 4). To investigate the expression of NF-κB as well as macrophage infiltration *in vivo*, immunohistochemistry was performed on paraffin sections of PC-3 tumor xenografts from nude mice. NF-κB and CD68 were highly expressed in mice treated with MCM, and were positively associated with tumor volume (Fig 4).

### SmsDX inhibits the nuclear translocation and activation of NF-κB induced via macrophage-conditioned medium in PCa cells

**Fig 5:**
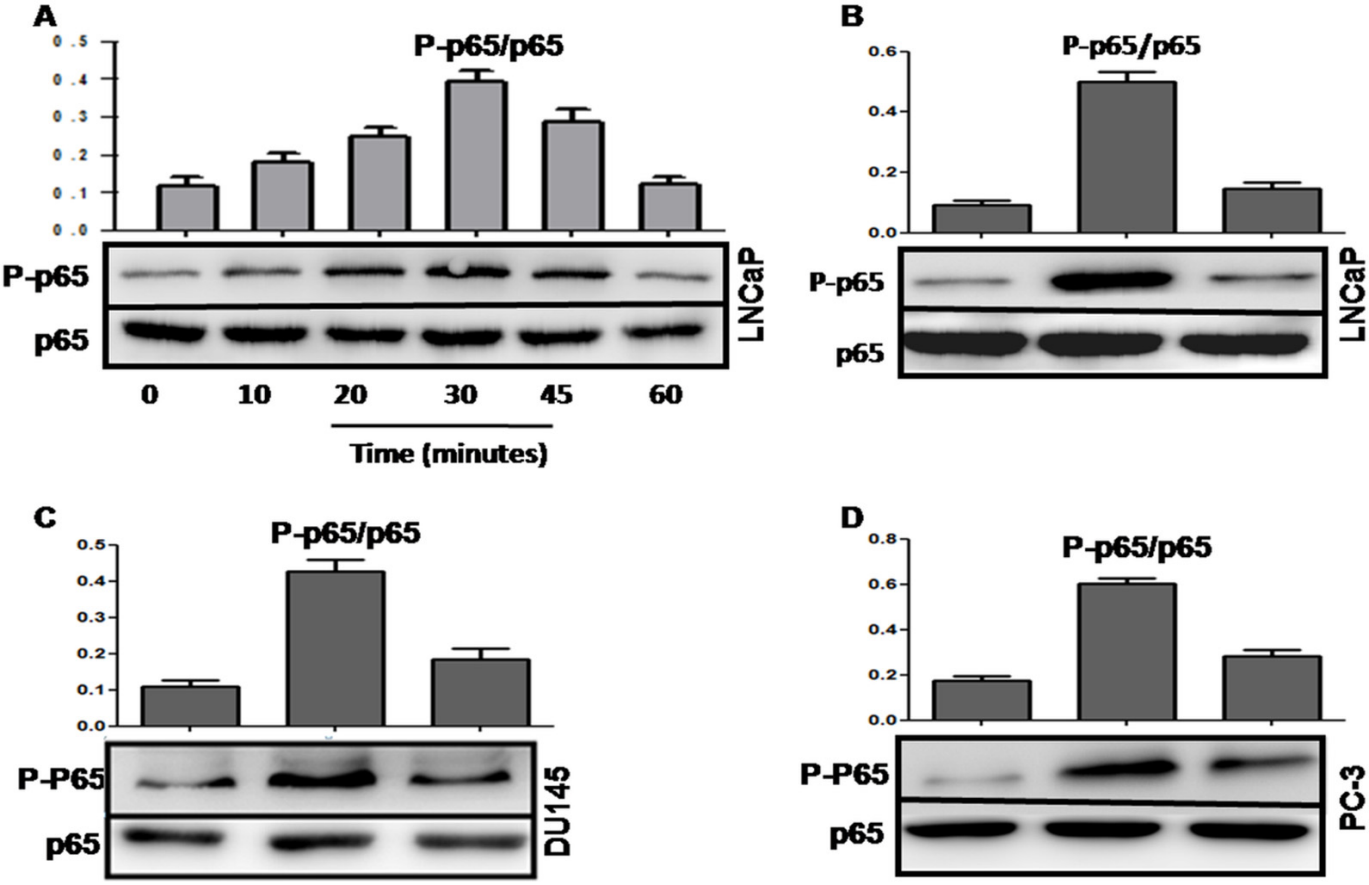
Effects of macrophage conditioned medium on the levels of phosphorylated NF-κB in the LNCaP, DU145 and PC-3 cells. (A) The levels of phosphorylated NF-κB in the LNCaP cells after exposure to MCM for different duration. (B-D) The levels phosphory and total NF-κB were analyzed via western blotting. The quantitation of the p-NF-κB/NF-κB ratio was determined via densitometry analysis.

NF-κB may represent a critical mechanistic link between inflammation and cancer, and its aberrant activation can promote tumor cell proliferation, tumorigenesis, migration and invasion[15,16]. We first observed the expression of phosphorylated P65, which modulates NF-κB transcription activity [27,28]. As shown in Fig 5, co-culture with MCM significantly up-regulated phosphorylated P65 in LNCaP cells, which was most strongly expressed when these cells were incubated with MCM for 30 min. Subsequently, we explored the effect of smsDX on the phosphorylation of P65. The up-regulated phosphorylation levels of P65 secondary to MCM treatment were effectively suppressed via smsDX in three PCa cell lines (Fig 5).

**Fig 6:**
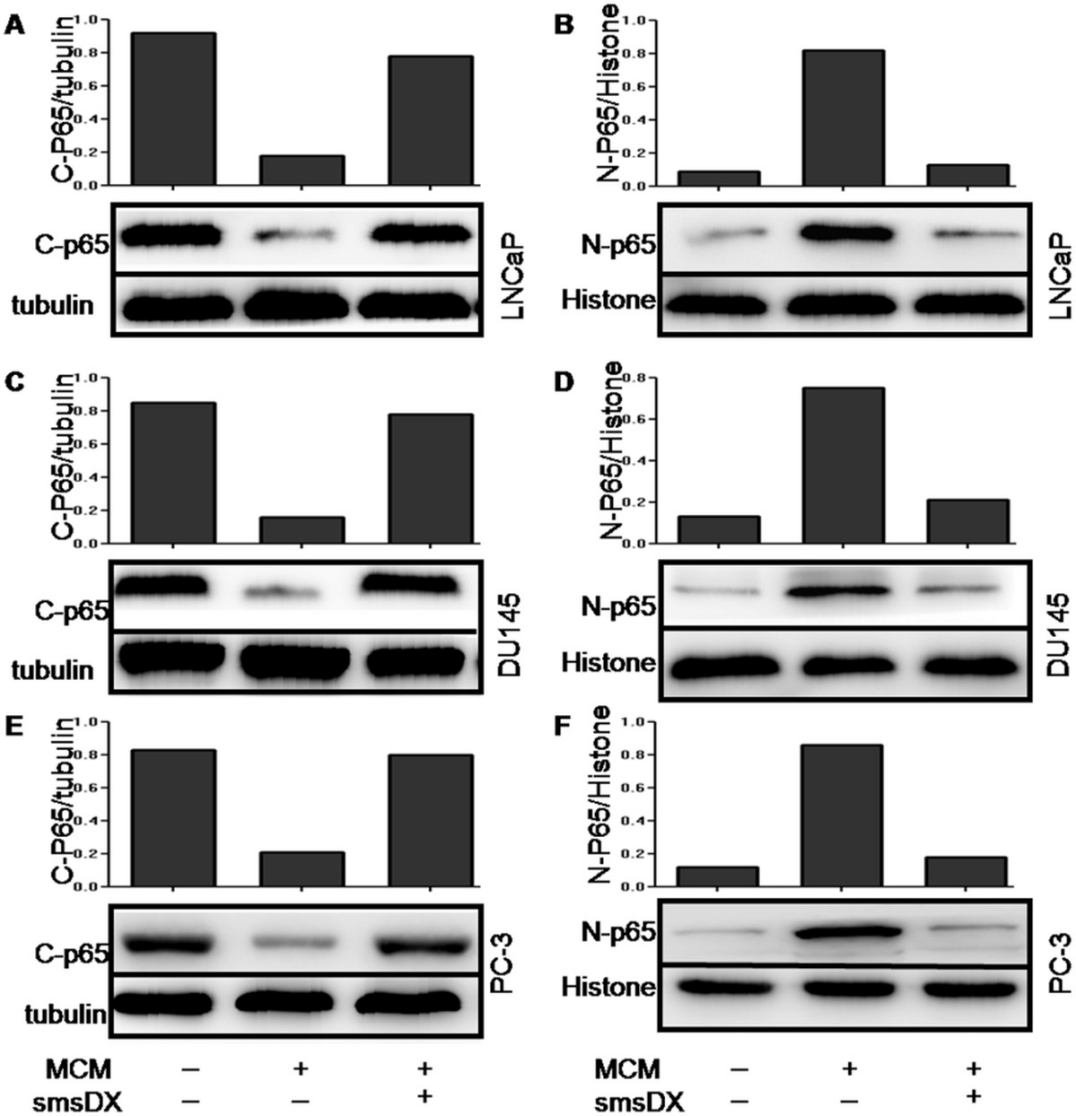
SmsDX inhibits the nuclear translocation of p65 induced by MCM in LNCaP, DU145 and PC-3 cells. (A, C, E)The expression of p65 in the cytoplasm was decreased after co-culture with MCM, smsDX attenuated this effect in LNCaP, DU145 and PC-3 cells. A-tubulin was chosen as the cytosolic protein loading control. (B, D, F) Co-culture with MCM increased the expression of nuclear p65, and treatment with smsDX weakened the effect of MCM. Histones were chosen as the nuclear protein loading control. The quantitation of the C-p65/ a-tubulin and N-p65/histone ratio was performed as densitometry analysis.

**Fig 7:**
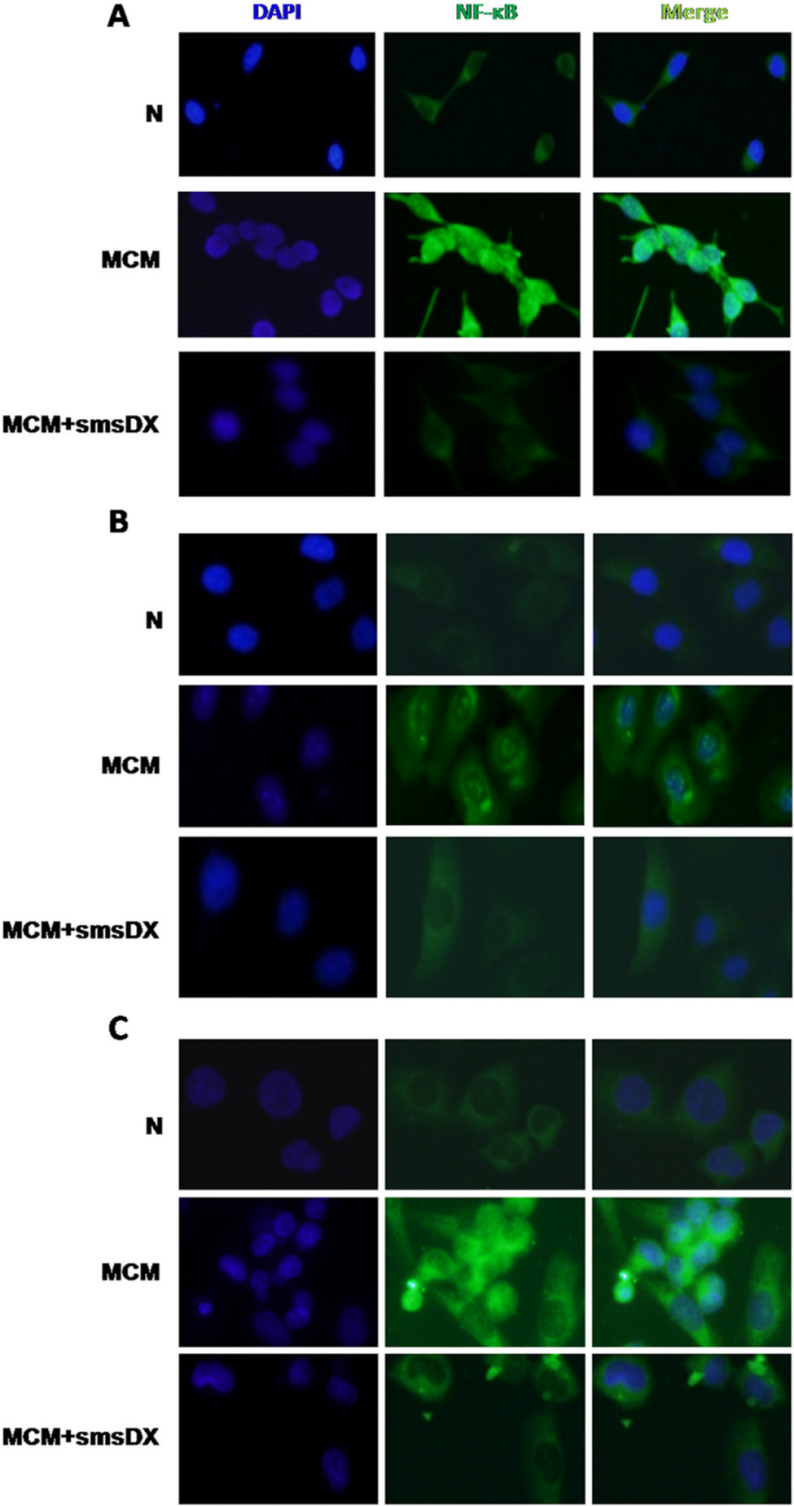
Effect of MCM and smsDX on the nuclear translocation of p65 via immunofluorescence. (A) Co-culture with MCM significantly promoted the nuclear accumulation of p65 in LNCaP cells. Treatment with smsDX inhibited p65 nuclear translocation. (B and C) The expression and translocation of p65 after exposure to MCM and treatment with smsDX in DU145 and PC-3 cells, respectively, were analyzed via immunofluorescence.

To further assess the effects of MCM and smsDX on the translocation of NF-κB, cytosolic and nuclear proteins were extracted, respectively. Western blot analysis revealed that MCM reduced the abundance of P65 in the cytoplasm of LNCaP cells (Fig 6). Moreover, increased levels of P65 were observed in the nucleus (Fig 6), indicating that NF-κB was activated. However, treatment with smsDX for 24 h dramatically inhibited the effect of MCM on NF-κB activation. Similar results were obtained in the DU145 (Fig 6) and PC-3 cell lines (Fig 6). The immunofluorescence results in Fig 7 further confirmed that, similar to the western blot assay, a significantly increase in the nuclear accumulation of P65 was observed after co-culture with MCM in LNCaP cells, and that smsDX dramatically inhibited P65 translocation. Similar results were observed in the DU145 and PC-3 cell lines (Fig 7).

## Discussion

The presence of leukocytes within tumors was first observed in the 19th century by Rudolf Virchow, this observation provided the first indication of a link between inflammation and cancer[29]. Epidemiological studies have recently shown that macrophages represent a major component of the host leukocyte infiltrate in the majority of malignant tumors[30]. However, the clinical significance of TAM infiltrates in prostate cancer remains controversial. Kiran el al reported that the mean number of TAMs was higher in prostate cancer than in benign tissue[31], whereas Norio et al reported the opposite result [32]. In the present study, we observed increased macrophage infiltration in prostate cancer and BPH with chronic inflammation compared with benign tissue. Increased TAM infiltration has also been reported to be associated with poor PCa progression[33]. Therefore, TAMs may represent a potential predictor of prostate cancer. Some previous studies have also revealed that M2-polarized THP-1 macrophages can promote tumor growth, invasion and metastasis[34,35]. In the present study, we found that TAM-like M2-type macrophages increased the migration and invasion of prostate cancer cells. Additionally, MCM promoted the tumorigenicity of PC-3 cells in a xenograft model in nude mice. Furthermore, the administration of smsDX attenuated the migratory and invasive potential of prostate cancer cells that was stimulated by M2-type macrophages.

We also observed that NF-κB expression in PCa and BPH with chronic inflammation specimens was significantly higher than that in BPH, this expression was positively correlated with macrophage infiltration. Therefore, we focused on the role of NF-κB in this process. Nuclear factor-κB (NF-κB) is an inducible dimeric transcription factor that belongs to the Rel/NF-κB family. The over-expression of genes coding for RelA/NF-κB factors has been observed in many tumors [36–39], and the dysregulation of NF-κB is believed to promote cell proliferation, motility, invasion and metastasis in most tumors including prostate cancer[40,41]. NF-κB is considered to be a major molecular producer of inflammation and can promote immunity by controlling the expression of genes involved in inflammation[42]. The present study demonstrated that macrophages activated NF-κB in PCa. This led to the progression of prostate cancer cells after co-culture with MCM. In the tumor microenvironment, cytokines from TAMs could activate NF-κB in tumor cells, thereby promoting tumorigenesis. Tumor cells in turn express CSF-1 and can further stimulate and recruit macrophages[43]. Therefore, there is a paracrine loop within TAM-enhanced tumor progression. And NF-κB plays an important role in the action of this loop.

Somatostatin and its analogues are neuroendocrine hormones and represent important mediators between the nervous and the immune systems. Our and other groups have found that these hormones can inhibit the proliferation and invasion of several tumor types[21,44]. More recently, significant experimental data have demonstrated that SMS analogues exhibit anti-inflammatory activity due to their inhibitory effect on the secretion of TNF-α, IL-8 and IL-1β[45,46]. However, to the best of our knowledge, there have been no studies on the effects of SMS analogues on the expression of NF-κB and biological behavior of PCa cells in an inflammatory microenvironment. In the present study, we found that smsDX substantially attenuated the macrophage-mediated stimulation of PCa cell proliferation and invasion by inhibiting the expression and activity of NF-κB *in vitro* and *in vivo*. Somatostatin receptor subtypes 1 and 2 have been detected in human macrophages[22], and immunoreactive somatostatin has been detected in the cytoplasm of macrophages. Therefore, we speculate that smsDX could bind with SSTR1 or 2 on the surface of macrophages and break the paracrine loop between the tumor and the inflammatory microenvironment, thereby alleviating the macrophage-stimulated progression of PCa.

Recently, metabolic disorders including obesity, type 2 diabetes, and atherosclerosis have been histologically viewed as chronic low-grade inflammation with significant macrophage infiltration [47,48]. Macrophages have been observed to be in close proximity with metabolic cells and were observed to possess an extensive transcriptional signature that regulated the metabolism of adipocytessuch as pyruvate carboxylase, PPAR-γ, apolipoprotein C1, etc[18]. In obese individuals, energy surplus or products from macrophages can activate NF-κB, further triggering macrophage recruitment, activation and the initiation and maintenance of an inflammatory reaction that ultimately results in insulin resistance[18]. In addition to exacerbating peripheral insulin insensitivity, NF-κB can also affect glucose metabolism and mitochondrial respiration, which is important for cell growth and oncogenesis[49]. Our previous study reported that smsDX can affect cellular metabolism and the mitochondrial function of PCa cells. Therefore, we speculate that smsDX may attenuate the effect of MCM on PCa cells via action upon cellular metabolism in addition to anti-inflammation.

Taken together, our study demonstrates that TAM can promote the development and progression of PCa, and that smsDX attenuates this effect by down-regulating NF-κB. These results indicate that smsDX may be a promising therapeutic drug for prostate cancer patients.

## Materials and Methods

### Ethics statement

All human specimens were obtained during therapeutic surgery. This study was performed using the samples over the shelves after the pathologic diagnosis. We telephoned all patients and obtained verbally informed consent. This is specifically approved by the Qilu Hospital Committee of Shan Dong University for Human Experiments. All animal care and experimental protocols complied with the Animal Management Rules of the Ministry of Health of the People’s Republic of China (document No 55, 2001). All animals were maintained at the Key Laboratory of Cardiovascular Remodeling and Function Research at the Qilu Hospital of Shandong University. This study was approved by the Qilu Hospital Committee of Shan Dong University for Human Experiments and by the Animal Care Committee of Shandong University.

### Patients and specimens

A total of 24 patients diagnosed with PCa and 45 patients diagnosed with BPH treated at the Department of Urology Surgery, Qilu Hospital of Shandong University between October 2010 and July 2012 were enrolled in this study. Thereinto, sixteen of the PCa patients underwent transrectal prostatic biopsy, and 8 patients underwent transurethral resection of prostate. Twelve of 45 benign prostatic hyperplasia (BPH) patients were diagnosed with histological chronic prostatic inflammation. The clinical parameters and pathologic classifications are presented and summarized in Table 1. None of patients had received preoperative adjuvant therapy. The diagnosis of each case was confirmed by two pathologists.

### PCa cell lines and cell culture

Human PCa cell lines (LNCaP, DU145 and PC-3) and THP-1 cells were purchased from the Type Culture Collection of the Chinese Academy of Sciences. All cells were grown in RPMI-1640 medium (Hyclone, Logan, UT) containing 10% fetal calf serum at 37°C in a 5% CO_2_ incubator. Becasue TAM represents a distinct M2-polarized macrophage population[50], THP-1 monocytes were differentiated into macrophages in RPMI media containing 5 ng/ml phorbol 12-myristate 13-acetate (Sigma Chemical Co., St. Louis, MO) for 48 h according to previously established protocols[51]. Then culture supernatants were filter-sterilized and stored at -20°C until ready for use.

### Immunohistochemical analysis

Immunohistochemistry was performed as described previously[52]. Briefly paraffin-embedded tissues were deparaffinized and rehydrated. After antigen retrieval, all specimens were exposed to primary antibody (CD68 1:50, P65 1:100) (Santa Cruz Biotechnology, Santa Cruz, CA) overnight at 4°C, and then incubated with biotin-conjugated secondary antibodies for 30 min at 37°C, followed by avidin–biotinylated peroxidase complex for 20 min at 37°C. Staining was executed with 3,3N-diaminobenzidine tertrahydrochloride and haematin. The protein expression level was assessed according to the percent of positively stained tumor cells and the staining intensity by two pathologists as described previously[53]. Briefly, the percentage of positive staining was scored as 0 (0–9%, negative), 1 (10%–25%, sporadic), 2 (26%–50%, focal) or 3 (51%–100%, diffuse), and the intensity as 0 (no staining), 1 (weak staining, visible at high magnification), 2 (moderate staining, visible at low magnification) and 3 (dark staining, strikingly positive at low magnification). The total immunostaining score was calculated with the value of percent positivity score × staining intensity score, which ranged from 0 to 9. The protein expression level was defined as following: “0+” (negative, score 0), “1+” (weakly positive, score 1–3), “2+” (positive, score 4–6), and “3+” (strongly positive, score7–9).

### Cell viability assay

Cell viability was determined via CCK8 assay. Briefly, LNCaP (6×10^3^ cells/well), DU145 (4×10^3^ cells/well) and PC-3 (4×10^3^ cells/well) cells in 200 μl of medium were seeded in 96-well plates. After 12 hours for adherence, the medium was replaced by conditioned medium from macrophages with/without smsDX (10nM). After 24–72 hours, the cultured cells were treated with 20 μl of Cell Counting kit-8 (Dojindo, Japan) and incubated at 37°C for an additional 4 hours according to the instructions of the manufacture. The absorbance was determined at 450 nm.

### Clone formation assay

LNCaP, DU145 and PC-3 cells at a density of 1×10^3^ cells/well were seeded in 6-well plates. After incubation overnight conditioned medium with/without smsDX (10nM) was added to the medium, and the cells were incubated at 5% CO_2_ and 37°C for two to three weeks. When visible clones formed on the plates, the cells were washed the clones with PBS and fixed with precooled methanol, and stained with 0.1% crystal violet for 20 min. The colonies containing more than 50 cells were counted under a microscope. The clone formation rate = (the clone number / the number of cell inoculation) × 100%.

### *In vitro* scratch assay

PCa cells were seeded in 24-well plates. After incubation for 24 hours, each well was manually scratched with a 200 μl pipette tip, washed with PBS three times and incubated with conditioned medium with/without 10nM smsDX. Images were taken again after 24 hour. The distance between two cell edges were analyzed using ImageJ software.

### Transwell invasion assay

Transwell invasion assay were performed as described previously[54]. Briefly, a total of 5×10^4^ cells suspended in 100 μl serum-free medium were added to the upper chambers of the transwell system (24 wells, 8-mm pore size; Corning Costar, Lowell, MA, USA) coated with 2 mg/ml Matrigel (BD Biosciences). TAM-conditioned medium with/without 10nM smsDX was then added to the lower chamber. After 24 hours, the non-invaded cells in the upper chamber were gently removed with a cotton swab whereas the cells attaching to the lower surface were fixed with precooled methanol and stained with 1% eosin. At least ten fields of each chamber were randomly selected and the cells were counted under the microscope.

For migration assay, the cells were seeded in the upper chambers without coated Matrigel. The rest of assay was performed as the Transwell invasion assay. After 24 hours, the cells on lower surface were also counted in at least ten randomly fields, then the cell number was analyzed statistically.

### Immunofluorescence assay

PCa cells were plated onto fibronectin-coated glass coverslips. After 24 h of incubation, the cells were rinsed with PBS, fixed in precooled methanol, and permeabilized with 0.2% Triton X-100. The fixed cells were preincubated in 1.5% normal goat serum and further incubated overnight with a primary antibody against P65 (1:100 dilution) at 4°C. After incubating with fluorescein-conjugated goat anti-rabbit IgG antibody at 37°C, the coverslips were the mounted on slides with PermaFluor Aqueous. Fluorescence was observed using a Zeiss Axioplan Universal microscope.

### Western blotting

The cells were lysed in RIPA buffer containing 1% protease inhibitors. To isolate cytoplasmic component from nuclear one, PCa cells were treated with a nuclear protein extraction kit (Beyotime Biotechnology, Wuhan, China) and centrifuged at 3400 r.p.m. for 10 min at 4°C. The cytoplasmic and nuclear components were then subjected to Western blotting. Equal amounts of proteins from each sample were separated via SDS-PAGE and transferred onto a PVDF membrane using a wet transfer apparatus (Bio-Rad, Hercules, CA, USA). The membranes were blocked with 5% non-fat milk for 2 h at room temperature and incubated with the primary antibodies overnight at 4°C. The membranes were subsequently exposed to the horseradish peroxidase-labeled secondary antibodies (1:2,000) for 1 h at room temperature, and reactivity was detected using an enhanced chemiluminescence detection system (Amersham, Pittsburg, PA). Protein levels were analyzed using ImageJ software.

### Tumor xenograft model

Pathogen-free 4-5-week-old BALB/c nude mice (weighing 19±2 g, SPF grade, certificate SCXK2011-0012) were purchased from the Department of Laboratory Animal Science at Peking University (Beijing, China). A total of 5×10^6^ of PC-3 cells were collected, mixed with Matrigel and injected subcutaneously in the flank of nude mice. The mice were randomly divided into three groups (5 per group). The mice were given of MCM with or without smsDX at a dose of 1 mg/kg/d via intraperitoneal injection for 4 weeks with weekly monitoring of the tumor size and body weight, while the control mice were given the same volume of normal saline. All of the mice were euthanized by using sodium pentobarbital 8 weeks after inoculation of the cancer cells and the tumors were collected.

### Statistical analysis

Data are mostly presented as the mean ± SD. SPSS software package (version 13.0; SPSS, Chicago, IL) was used for all statistical analysis. Significant differences between treatment and control values were analyzed using Student’s two-tailed t-test or one-way analysis of variance wherever appropriate. Differences were considered to be statistical significantly when p<0.05. Each variable was tested twice and the experiment was repeated three times.
